# Virtual lab of artificial intelligence agents accelerating nanobody design against SARS-CoV-2 variants

**DOI:** 10.7150/ijbs.126093

**Published:** 2026-01-01

**Authors:** Hakjin Kim, Taeho Kwon, Sun-Uk Kim, Seon-Kyu Kim

**Affiliations:** 1 Quantum AI Bio Research Laboratory (KJQI-JQL), In Quantio, Gene on Biotech, Daejeon 35229, Republic of Korea.; 2 Advanced Bioconvergence Department, Department of Bioscience, KRIBB School, Korea National University of Science and Technology (UST), Daejeon 34113, Republic of Korea.; 3 Quantum AI Bio Research Laboratory (KJQI-JQL), Futuristic Animal Resource and Research Center, Korea Research Institute of Bioscience and Biotechnology (KRIBB), Cheongju, Chungbuk 28116, Republic of Korea.; 4 Quantum AI Bio Research Laboratory (KJQI-JQL), Genomic Medicine Research Center, Korea Research Institute of Bioscience and Biotechnology (KRIBB), Daejeon 34141, Republic of Korea.

**Keywords:** artificial intelligence, virtual lab, nanobody design, SARS-CoV-2 variants, large language models

## Abstract

Artificial intelligence (AI)-driven research frameworks are reshaping the boundaries of biomedical discovery. The Virtual Lab exemplifies this transformation, assembling large language model (LLM) agents into coordinated scientific teams functioning as investigators, specialists, and critics. The system autonomously designed, scored, and refined nanobody candidates against emerging SARS-CoV-2 variants, and subsequently validated them experimentally. These findings demonstrate how AI can move beyond prediction and retrieval to serve as an active collaborator in hypothesis generation, experimental design, and translational application. As technology continues to advance, the convergence of artificial intelligence and quantum computing is expected to give rise to a new era of Quantum AI enabled biomedical research. This integration will accelerate discovery speed, enhance precision, and foster interdisciplinary collaboration, opening unprecedented opportunities for data-driven innovation in the life sciences.

## Introduction

Recent advances in artificial intelligence (AI) have introduced the concept of Virtual Labs digital research environments where autonomous agents collaborate to accelerate biomedical discovery 1. In the Virtual Lab, each agent was assigned a distinct role that mirrored the responsibilities of human scientists, enabling structured collaboration and critical oversight 1. A principal investigator agent set agendas and synthesized discussions; domain specialists, such as immunologists or computational biologists, contributed expert reasoning; and a scientific critic carefully examined assumptions and identified errors. The agents interacted in a structured manner, exchanging ideas, challenging each other's reasoning, and gradually converging on research strategies. Human involvement was deliberately minimized and limited to providing high-level oversight and performing laboratory experiments, accounting for only approximately 1% of the total content generated.

To demonstrate the potential of this system, the researchers applied Virtual Lab to the urgent challenge of designing nanobody binders against newly emerging SARS-CoV-2 variants. Variants such as JN.1 and KP.3, descendants of Omicron lineages, carry multiple mutations in the receptor-binding domain of the spike protein that allow them to evade several existing neutralizing antibodies. The rapid pace of viral evolution has consistently outstripped traditional therapeutic pipelines, which usually require extended timelines for antibody discovery and optimization.

**Figure 1:**
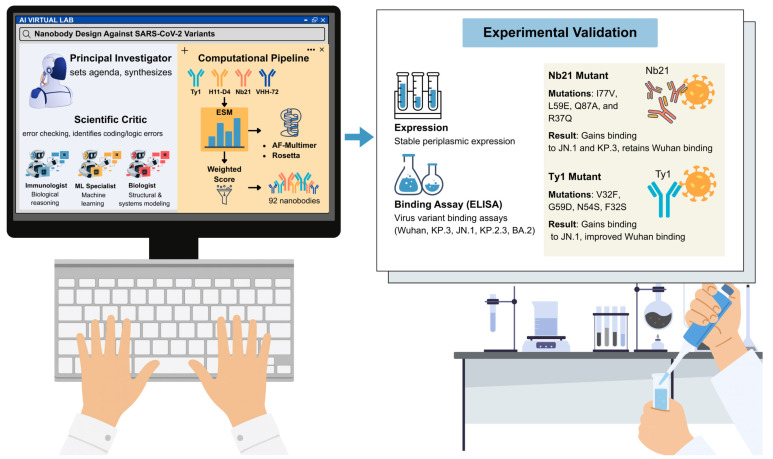
**AI Virtual Lab for new SARS-CoV-2 nanobody design and experimental validation.** Virtual Lab organizes large language model (LLM) agents into coordinated scientific roles, including a principal investigator (PI) that sets agendas and synthesizes strategies, specialist agents such as immunologists, machine learning (ML) experts and biologists, and a scientific critic that performs error checking and logic verification. These agents collectively construct a computational pipeline for nanobody engineering. Starting from known nanobody scaffolds (Ty1, H11-D4, Nb21, and VHH-72), mutations are evaluated using the ESM protein language model, modeled with AlphaFold-Multimer, and refined with Rosetta. Candidate designs are ranked through a weighted scoring system, yielding 92 nanobody sequences. Experimental validation confirms high levels of periplasmic expression and identifies mutants with binding activity against immune-evasive SARS-CoV-2 variants. Notably, Nb21 (I77V, L59E, Q87A, and R37Q) gained binding to JN.1 and KP.3, while Ty1 (V32F, G59D, N54S, and F32S) newly acquired binding to JN.1. This integrated AI-to-experiment pipeline demonstrates the ability of AI multi-agent systems to autonomously generate and validate functional therapeutic molecules.

As shown in Fig. 1, the Virtual Lab autonomously constructs a sophisticated design pipeline integrating multiple complementary approaches. Mutations were first analyzed using the evolutionary-scale prediction of atomic-level protein structure with a language model (ESM) to estimate evolutionary plausibility and stability 2. High-scoring candidates were then modeled with AlphaFold-Multimer to predict structural compatibility with the viral receptor-binding domain, while Rosetta optimization refined energetic stability and binding free energies 3. These layers, spanning sequence plausibility, structural modeling, and energetic optimization, were consolidated into a weighted scoring metric that guided candidate selection.

From this iterative process emerged 92 candidate nanobodies, which represented a complete in silico-to-in vitro discovery pipeline developed entirely by AI agents 1. Through cycles of mutation, modeling, and scoring, Virtual Lab reproduced the type of design-test-refine loop, normally requiring extensive human coordination across disciplines, autonomously within only a few days.

A decisive step occurred when computational predictions were experimentally tested. More than 90% of the candidates were expressed as soluble proteins in *Escherichia coli*, indicating that the proposed mutations did not destabilize the nanobody scaffold. Binding assays revealed that these two candidates acquired novel activities. An Nb21 mutant carrying the substitutions I77V, L59E, Q87A, and R37Q gained binding activity against both JN.1, and KP.3, while retaining a strong affinity for the ancestral Wuhan strain. A Ty1 mutant incorporating substitutions V32F, G59D, N54S, and F32S gained binding capacity to JN.1, whereas the parental Ty1 showed no activity and demonstrated improved recognition of the Wuhan receptor-binding domain (Fig. 1) 1.

These results highlight the potential of the Virtual Lab to significantly shorten therapeutic discovery timelines and broaden access to advanced design strategies beyond resource-rich research centers. By compressing processes that normally take months to weeks, Virtual Lab illustrated the possibility of responding to viral evolution at a speed more closely matching pathogen dynamics. By embedding expertise in AI agents, the system also reduced the barriers for smaller institutions to participate in sophisticated interdisciplinary projects, suggesting a path toward democratized access to therapeutic design.

The analysis of the AI agent Virtual Lab (principal investigator agent, domain specialist agents such as immunologist or computational biologist, and scientific critic agent) discussions provides deeper insights into the importance of structured governance within autonomous research environments. Agent teams with clearly defined specialist roles and dedicated critic agents generated coherent and biologically grounded strategies. For instance, they favored optimizing existing nanobody scaffolds rather than pursuing less practical de novo designs, consistent with earlier human-led nanobody optimization studies 4. The critic agents played a central role in detecting and correcting errors in coding, logic, and reasoning. In contrast, groups composed of generic, non-specialized agents engaged in redundant debates and often failed to converge on effective strategies. These findings indicate that the quality of AI-driven scientific reasoning depends not only on the underlying language models but also on the organizational design that governs their interaction mirroring the dynamics observed in human research teams.

The implications of this study extend beyond methodology to scientific philosophy. Virtual Lab reframes artificial intelligence from a passive tool to an active collaborator capable of hypothesis generation, debate, and discovery. By engaging in structured reasoning and critical evaluation, agents challenge conventional assumptions about creativity and knowledge production. They acted not simply as calculators or assistants but also as participants in scientific discourse, raising important questions about authorship, accountability, and collaboration in the age of digital intelligence.

Despite these advances, the limitations of this study need to be addressed in future studies. The current LLM has knowledge cutoffs that prevent it from incorporating the most recent methods or findings without external augmentation. The quality of their output is highly dependent on how agendas are formulated, meaning that a careful design of instruction and discussion flow is required in a manner comparable to experimental design in traditional science. There is also a persistent risk of generating incorrect or misleading content, making rigorous human oversight indispensable. Furthermore, while binding assays confirmed that certain candidates could attach to variant receptor-binding domains, further evaluation of the neutralization potency, pharmacokinetics, immunogenicity, and safety is required before clinical applications can be considered 1.

Even with these constraints, Virtual Lab points toward a future in which AI-human collaborations play an integral role in biomedical research. Platforms that continuously monitor viral genomic data worldwide, autonomously propose updated therapeutic candidates, and integrate them into high-throughput organoid testing for rapid validation can be envisioned. Beyond *in vitro* validation, Virtual Lab's multi-agent framework could be extended to orchestrate animal model experimentation, enabling the autonomous design and optimization of *in vivo* methodologies. This is crucial for comprehensive preclinical evaluation and assessment of factors such as neutralization potency, pharmacokinetics, immunogenicity, and safety before therapeutic candidates can advance toward clinical applications. Similar frameworks can also be adapted to oncology, where antibodies are designed to target tumor-specific neoantigens or other important biomedical domains. Furthermore, professional medical agent teams can be established by leveraging the Virtual Lab architecture for structured debate and critical evaluation. These teams, composed of specialized AI agents reflecting diverse medical expertise, such as cardiology, oncology, and radiology, can collaborate in a virtual setting to offer comprehensive patient consultations, aid in differential diagnosis, and develop personalized treatment plans, thereby broadening access to specialized medical knowledge and reducing diagnostic barriers.

This study should be regarded as a proof-of-principle for a new model of science in which discovery is redefined as a structured dialogue between digital intelligence and experimental biology. This study is unique in that it transforms AI from a passive tool into a true collaborator. Equally significant is its demonstration of compressed timelines and democratized access to expertise, showing how advanced design strategies can be extended beyond resource-rich centers. Most importantly, the essential message is that structured AI-human teams can generate authentic, experimentally validated biomedical discoveries. Rather than retaining a distant vision, Virtual Lab offers a practical pathway toward more agile responses to rapidly evolving health threats. With continued refinement, it may become a cornerstone of translational medicine in the coming decade. As computing paradigms advance, the convergence of artificial intelligence and quantum technologies is expected to give rise to a new era of Quantum AI-enabled Virtual Labs, where discovery processes will become even faster and more precise, further accelerating the pace of biomedical innovation 5. Such a convergence would make discovery processes even faster and more precise, further accelerating the pace of biomedical innovation and expanding the frontiers of translational science.

## Abbreviations

AI: Artificial intelligence
LLM: Large language model
PI: Principal Investigator
Quantum AI: Quantum artificial intelligence

## References

[1] Swanson K, Wu W, Bulaong NL, Pak JE, Zou J (2025). The Virtual Lab of AI agents designs new SARS-CoV-2 nanobodies. Nature.

[2] Lin Z, Akin H, Rao R, Hie B, Zhu Z, Lu W (2023). Evolutionary-scale prediction of atomic-level protein structure with a language model. Science.

[3] Jumper J, Evans R, Pritzel A, Green T, Figurnov M, Ronneberger O (2021). Highly accurate protein structure prediction with AlphaFold. Nature.

[4] Linsky TW, Vergara R, Codina N, Nelson JW, Walker MJ, Su W (2020). De novo design of potent and resilient hACE2 decoys to neutralize SARS-CoV-2. Science.

[5] Ahart J (2025). AI helps assemble 'brain' of future quantum computer. Nature.

